# Low methane phenotype of feedlot steers changes throughout production and depends on diet

**DOI:** 10.1093/jas/skag104

**Published:** 2026-05-20

**Authors:** Melissa S Williams, Stephanie A Terry, Karen A Beauchemin

**Affiliations:** Lethbridge Research and Development Centre, Agriculture and Agri-Food Canada, Lethbridge, Alberta T1J 4B1, Canada; Lethbridge Research and Development Centre, Agriculture and Agri-Food Canada, Lethbridge, Alberta T1J 4B1, Canada; Lethbridge Research and Development Centre, Agriculture and Agri-Food Canada, Lethbridge, Alberta T1J 4B1, Canada

**Keywords:** beef steers, enteric methane, GreenFeed, silage source, variation

## Abstract

A better understanding of the variability in methane (**CH_4_**; g/day) emissions from beef cattle throughout production is necessary to implement mitigation programs. One hundred Angus crossbred steers were fed diets including either barley silage (**BS**) or corn silage (**CS**) throughout a backgrounding (**BG**) and finishing (**FN**) phase feedlot study. The objectives were to characterize the variability in methane yield (**CH_4_Y**; g/kg dry matter intake) among and within steers during BG and FN phases of production, assess whether the variability in CH_4_Y differs by silage type, examine the stability of CH_4_Y ranking (**Low**, **Intermediate**, or **High**) of steers within and across phases, and evaluate relationships between range of CH_4_Y, CH_4_ production, and feeding behavior. Cattle were fed a BG diet (70% silage and 22%–26% barley grain on a DM basis) for 121 d followed by a FN diet (15% silage and 77%–78% barley grain on a DM basis) for 158 d, with each phase comprised of two measurement periods. Steers were assigned to one of the silage sources for the entirety of the experiment with n = 50 each. Enteric CH_4_ was measured using GreenFeed emissions monitoring systems (C-Lock Inc., USA) for 2 wks/period in BG and 2–3 wks/period in FN. Steers were ranked based on their CH_4_Y (**MYR**) in each period and phase and categorized as Low (<0.5 SD from the mean), Intermediate (within ±0.5 SD of the mean), or High (>0.5 SD of the mean) emitters. Statistical analysis was performed within a period or phase using PROC GLIMMIX of SAS as a mixed model with fixed effects of MYR, silage, MYR×silage, and period or phase. Steer was the experimental unit, and the random effect accounted for individual variability. Rankings of individuals were compared across periods and phases to determine whether rankings changed over time. The CH_4_Y of steers ranked Low in period 1 were not different from Intermediate steers in period 2 of BG, with both being lower than High steers (*P *< 0.01). Similarly, steers ranked as Low in BG overall were not different from those ranked as Intermediate in FN for CH_4_Y (*P *< 0.01). During FN, Low and Intermediate steers differed in period 2, whether using period 1 or 2 ranking (*P *< 0.001). During BG, Low and Intermediate steers had a lower (*P *< 0.01) proportion of caproate in rumen fluid than High steers, and cattle fed BS had increased (*P *≤ 0.01) propionate and valerate and decreased (*P *≤ 0.01) acetate and NH_3_-N. During FN, Low steers had lower (*P *= 0.03) acetate and higher (*P *≤ 0.04) propionate and isobutyrate proportions than high steers. Steers were more likely to change MYR during BG (56%) and from BG to FN (42%), compared to during FN (20%). During BG, 14% of BS and 71% of CS steers ranked as low in period 1 changed rank in period 2. When steers were ranked as low during BG, 43% (BS) and 57% (CS) had a different MYR in FN. Pearson correlation coefficients revealed a moderate to strong positive relationship between the CH_4_Y range and CH_4_ and CH_4_Y during FN for both silage sources (*P *< 0.05). For CS steers, feed intake decreased as the CH_4_Y range increased (*P *< 0.001; r = -0.61). In conclusion, the variability in CH_4_Y was present throughout BG and FN, and MYR was most likely to change within BG and across phases. Therefore, these results suggest that selecting steers for their MYR based on a short-term measurement may not reflect lifetime MYR within and across phases and diets.

## Teaser

This study reveals that methane emissions from beef cattle vary significantly over time, especially when fed high forage diets and across dietary transitions. Repeated measurements over time and across diets are needed to identify consistent low-emitting cattle for genetic selection of low methane producing feedlot cattle.

## Introduction

Methane (**CH_4_**), a potent greenhouse gas, is a byproduct of ruminant enteric fermentation. Mitigation of enteric CH_4_ emissions is crucial for minimizing global warming. In addition to its contribution to climate change, enteric CH_4_ constitutes a loss of 2% to 12% of gross energy intake in cattle (Johnson and Johnson 1995), which can potentially diminish production efficiency. Decreasing CH_4_ emissions from cattle offers a dual benefit: a decreased carbon footprint of cattle production and the potential for enhanced feed efficiency.

The amount of CH_4_ produced by ruminants depends on numerous factors, including dry matter intake (**DMI**), diet composition, rumen microbiome, host genetics, animal physiology, and animal behavior. Thus, even when animals are fed a common diet, CH_4_ emissions are variable. Roehe et al. (2016) found that CH_4_ emissions measured using respiration chambers from 34 beef calves fed either forage or concentrate-based diets for 56 d were highly variable between animals (152–333 and 78–233 g/d for calves on forage and concentrate diets, respectively). Using GreenFeed emissions monitoring systems (**GEMS**; C-Lock Inc., Rapid City, SD, USA), cattle fed high-concentrate feedlot diets had a between-animal coefficient of variation of 17.3% over 2 wks across three periods (Beauchemin et al. 2022). In that study, period was found to be significant for CH_4_ emissions, with CH_4_ production (g/d) and CH_4_ yield (**CH_4_Y**; g/kg DMI) being greater in period 2 compared to periods 1 and 3, indicating variation throughout the feeding period; however, individual animal variability over time was not considered.

Methane production is strongly and positively correlated with DMI and fiber concentration of the diet (Grainger et al. 2007; Fuller et al. 2020); however, Beauchemin et al. (2022) reported that cattle ranked as low or high CH_4_ emitters had similar DMI. It has been suggested that the feeding behavior of cattle, particularly in terms of meal duration and frequency, affects CH_4_ emissions, although this relationship is not well-documented (Johnson et al. 1994; De Mol et al. 2024). Another potential factor contributing to variability is the ruminal microbial community composition and the resulting volatile fatty acid (**VFA**) profile, electron flow, and ultimately CH_4_ production (Wolin et al. 1997; Zhou et al. 2010; Ghosal et al. 2012).

Heritability in CH_4_ emissions among animals is well-documented, with estimates for cattle ranging from 0.25 to 0.42 (Hayes et al. 2016; Manzanilla-Pech et al. 2016; Brito et al. 2018; Souza et al. 2024). Genetic selection of low-emitting animals has the potential to make a permanent and cumulative reduction in CH_4_ emissions. A cornerstone of such breeding programs is the measurement of the CH_4_ phenotype of animals. Measuring CH_4_ production for a large group of animals is costly and labor-intensive; thus, assessments are rarely repeated over time and across diets. However, there is uncertainty regarding the permanence of an animal’s CH_4_ ranking over time and across diets.

Therefore, the objectives of this experiment were to: 1) characterize the variability in CH_4_Y of steers during backgrounding (**BG**) and finishing (**FN**) phases of production, 2) assess whether the variability in CH_4_Y differs between steers fed a barley silage (**BS**) or corn silage (**CS**) based diet, 3) examine the stability of CH_4_Y ranking (Low, Intermediate, or High) within and across BG and FN production phases, and 4) evaluate the influence of feeding behavior and CH_4_ production measurements on the variability of CH_4_Y.

## Materials and methods

This experiment was conducted at the Lethbridge Research and Development Centre of Agriculture and Agri-Food Canada (Lethbridge, Alberta, Canada) according to the guidelines of the Canadian Council on Animal Care (CCAC 2009) and was approved by the Institutional Animal Care Committee (#ACC1927).

### Experimental design

The experiment used a randomized complete block design, with initial body weight (**BW**) as a blocking factor and steer as the experimental unit. One hundred single-sourced Angus crossbred beef steers were weighed on two consecutive days and stratified into light (258 ± 7.1 kg) and heavy (294 ± 17.4 kg) groups, with 50 steers per group. Within each weight block, steers were randomly allocated into two feedlot pens (25 animals/pen) and assigned a silage source (BS or CS). Each pen was equipped with 5 GrowSafe (Vytelle Inc., Lenexa, Kansas, USA) feed bunks, measuring daily individual animal feed intake and feeding behavior. The pens were bedded with straw as needed.

Upon arrival, steers were administered a Component TE-100 implant (100 mg trenbolone acetate (TBA), 10 mg of estradiol, and 29 mg of tylosin tartrate; Elanco Animal Health, Division Eli Lilly Canada Inc., Guelph, ON, Canada), vaccinated with with Ultrabac 7/Somubac (Clostridium vaccine; Zoetis Canada Inc., Kirkland, QC, Canada) and Pyramid FP5 (infectious bovine rhinotracheitis and bovine viral diarrhea vaccine; Boehringer Ingelheim Ltd., Burlington, ON, Canada), and treated with Bovimectin Pour-on (5 mg of ivermectin/mL; 500 μg of ivermectin per kg of BW; Vetoquinol N.-A. Inc., Lavaltrie, QC, Canada). At the start of the FN, steers were implanted for a second time with Component TE-200 (200 mg TBA, 20 mg of estradiol, and 29 mg of tylosin tartrate; Elanco Animal Health, Division Eli Lilly Canada Inc., Guelph, ON, Canada).

The study consisted of a BG phase (121 d), a transition period (28 d), and an FN phase (158 d). Enteric CH_4_ measurements began in BG after a 2-wk adaptation period (d −14 to 0) on the BG diet and were performed over two 4-wk periods (experiment d 1–56). For FN, CH_4_ measurements were conducted at the end of the phase over two consecutive 5-wk periods (experiment d 237–307) after cattle were on the FN diet for 98 d. Cattle were rotated through the pens on a weekly basis throughout the experiment so that each group had 1 wk with access to the GEMS, followed by one without. Thus, within a period, CH_4_ was measured over 2 wks during BG and 3 wks during FN for each animal. During BG, steers were fed a diet of 70% silage (BS or CS), 22% (CS) or 26% (BS) barley grain, and 8% (CS) or 4% (BS) mineral/vitamin supplements to formulate for similar crude protein (**CP**) and gross energy between diets. When the steers reached approximately 400 kg, they were transitioned to the FN diet. During FN, the calves were fed a diet of 78% dry-rolled barley grain, 15% silage (BS or CS), and 7% mineral/vitamin supplement. The diets were formulated to meet or exceed the nutrient requirements of growing and finishing beef steers (NASEM 2016) and are presented in Table 1. The study continued until the steers attained a live BW of approximately 700 kg, and then they were shipped to a commercial abattoir for harvesting.

**Table 1:** Composition of total mixed ration (TMR) and GreenFeed pellet for barley silage and corn silage diets.

	Backgrounding		Finishing	
Ingredients	Corn silage	Barley silage	Corn silage	Barley silage
**Treatment TMR (DM Basis)**				
**Corn silage**	70		15	
**Barley silage**		70		15
**Barley grain, dry rolled**	22.07	26.48	77.43	78.54
**Vitamin mineral premix^1^**	7.93	3.52	7.57	6.46
**TMR composition, % DM**				
**DM, %**	39.7 ± 2.23	46.0 ± 1.49	75.2 ± 3.88	76.7 ± 2.97
**OM**	94.3 ± 0.23	92.5 ± 0.23	95.5 ± 0.47	93.9 ± 0.47
**CP**	12.8 ± 0.77	11.9 ± 0.77	12.3 ± 1.55	12.8 ± 1.55
**Starch**	28.7 ± 4.06	26.5 ± 4.06	40.6 ± 8.11	42.9 ± 8.11
**NDF**	39.7 ± 3.26	38.6 ± 3.26	25.6 ± 6.53	30.1 ± 6.53
**Gross energy, Mcal/kg**	4.85	4.82	4.82	4.83
**Green feed pellet (DM Basis)**				
**Canola meal**	7.41		3.94	
**Limestone**	0.84		0.84	
**Salt**	0.15		0.15	
**Molasses**	15.18		15.14	
**Barley ground**	45.14		63.97	
**Canola oil**	0.71		0.71	
**Beet pulp**	30.57		15.25	

1 Vitamin and mineral premix was formulated to meet the requirements of animals according to NASEM (2016) and to create isoenergetic and isonitrogenous diets including; canola meal, ground barley grain, limestone, urea, salt, a feedlot vitamin and mineral supplement, molasses and canola oil.

### Feed intake

Individual steer feed intake and feeding behavior were measured using GrowSafe feed bunks throughout the BG and FN phases. The DMI was calculated by summing the intakes of total mixed ration (**TMR**) at each feeding event during each 24-h cycle and corrected for the dry matter (**DM**) concentration of the TMR. The eating behavior of individual steers was based on the information from the GrowSafe feeding system. The behaviors were described as feeding time (total amount of time spent each day with their head in the feed bunks), feeding rate (amount of DM consumed per day divided by feeding time), number, size, and length of meals, and intermeal duration, as described by Koenig et al. (2020). A meal was defined as a visit to the bunk followed by an absence from the bunk of 300 s or more, and meal size was the amount of feed DM consumed per meal.

Samples of silages and TMR were collected weekly, and samples of barley grain, vitamin/mineral supplement, and GEMS pellets once per period. To determine DM, samples of ingredients and TMR were dried in a forced-air oven at 55 °C for 48 h. Weekly silage DM content was used to adjust the ingredient proportions of the diets if DM deviated by >3% from the average. The weekly samples collected were pooled by 3-wk periods after being ground to 4 mm using a Wiley mill (Thomas Scientific, Swedesboro, NJ). All samples were then ground to 1 mm and stored at −20 °C until analyzed. The analytical DM content of the ground samples was determined by drying at 135 °C for 2 h (AOAC 2005; method 930.15). Organic matter (**OM**) was determined by ashing the samples in a muffle furnace at 550 °C for 5 h. The neutral detergent fiber (**NDF**) concentrations were determined using an Ankom A200 fiber analyzer (Ankom Technology, Macedon, NY) with heat-stable amylase and sodium sulfite. Samples were ground to a fine powder using a ball grinder (Mixer Mill MM2000; Retsch GmbH, Haan, Germany) before determining nitrogen and starch concentrations. The nitrogen concentration [CP = nitrogen × 6.25] was determined by flash combustion, gas chromatographic separation, and thermal conductivity detection (AOAC 2005, method 990.03; Carlo Erba Instruments, Milan, Italy). Starch concentration was determined by enzymatic hydrolysis and colorimetric glucose detection, as described by Koenig et al. (2013). Gross energy was determined in an automatically controlled Parr adiabatic oxygen bomb calorimeter (Parr Instrument Co., Inc., Moline, IL; Model 6200 Calorimeter).

### Methane measurement and ranking

To measure individual enteric CH_4_ emissions, two of the four pens were equipped with a GEMS system (C-Lock Inc., Rapid City, SD, USA). Steers were rotated between the pens every week, allowing for biweekly gas emission measurement for each animal. Moving the steers throughout the two pens controlled for pen effects and enabled CH_4_ to be measured over the study using a single GEMS system per silage source. The GEMS system allowed steers to move freely (in and out of the system), and gases were measured when the head of the steer was in the “head chamber” unit, as determined by the proximity sensor. The principles of measuring enteric CH_4_ emissions using the GEMS system in a pen of animals have been published in detail elsewhere (Alemu et al. 2021). The infrared gas analyzer and the air flux sensor were calibrated weekly, and the air filter was cleaned and changed regularly whenever flow readings were less than 30.0 L/s.

To entice steers to enter the GEMS system, pellets (Table 1) were dispensed as bait from an overhead hopper when their head was near the head chamber sensor. The animals could visit the system anytime during the day, with pellets set to drop once every 30 s while the head remained in the chamber to a maximum of six pellet drops per visit (last drop at the 3-min mark) or 4 h window (36 pellet drops per 24 h) to restrict the amount consumed. Pellet consumption (DM basis) was calculated using the average pellet drop weight (measured on a bi-weekly basis), visit duration, and the number of visits per day, correcting for the DM of the pellet. The DMI reported in this manuscript is a sum of both the TMR and pellet intake on a DM basis.

All GEMS data were downloaded from the C-Lock server. The data were pre-processed by removing negative values, data where a steer could not be identified, and visits with a duration of less than 3 mins. The remaining data were examined by period within phase. The fluxes were compiled into six 4-h time blocks and averaged within each time block (Manafiazar et al. 2016). The mean daily CH_4_ production was calculated by averaging overtime blocks to ensure the entire 24-h cycle of emission was represented. As visitation to the GEMS system and the diurnal pattern of CH_4_ relative to feeding are not evenly distributed throughout the 24-h cycle, using the time block method provides a more accurate representation of diurnal CH_4_ production, especially when visitation frequency is low (Jonker et al. 2014; Manafiazar et al. 2016). The energy lost by CH_4_ production (**Ym**) was calculated as a percentage of gross energy intake (**GEI**). Steers with < 10 total visits or had visits in < 5 time blocks were omitted from the period analysis (Terry and Beauchemin 2025). A total of 50 steers met the criteria for final analysis, with n = 23 for BS-fed steers and n = 27 for CS-fed steers.

**Figure 1:**
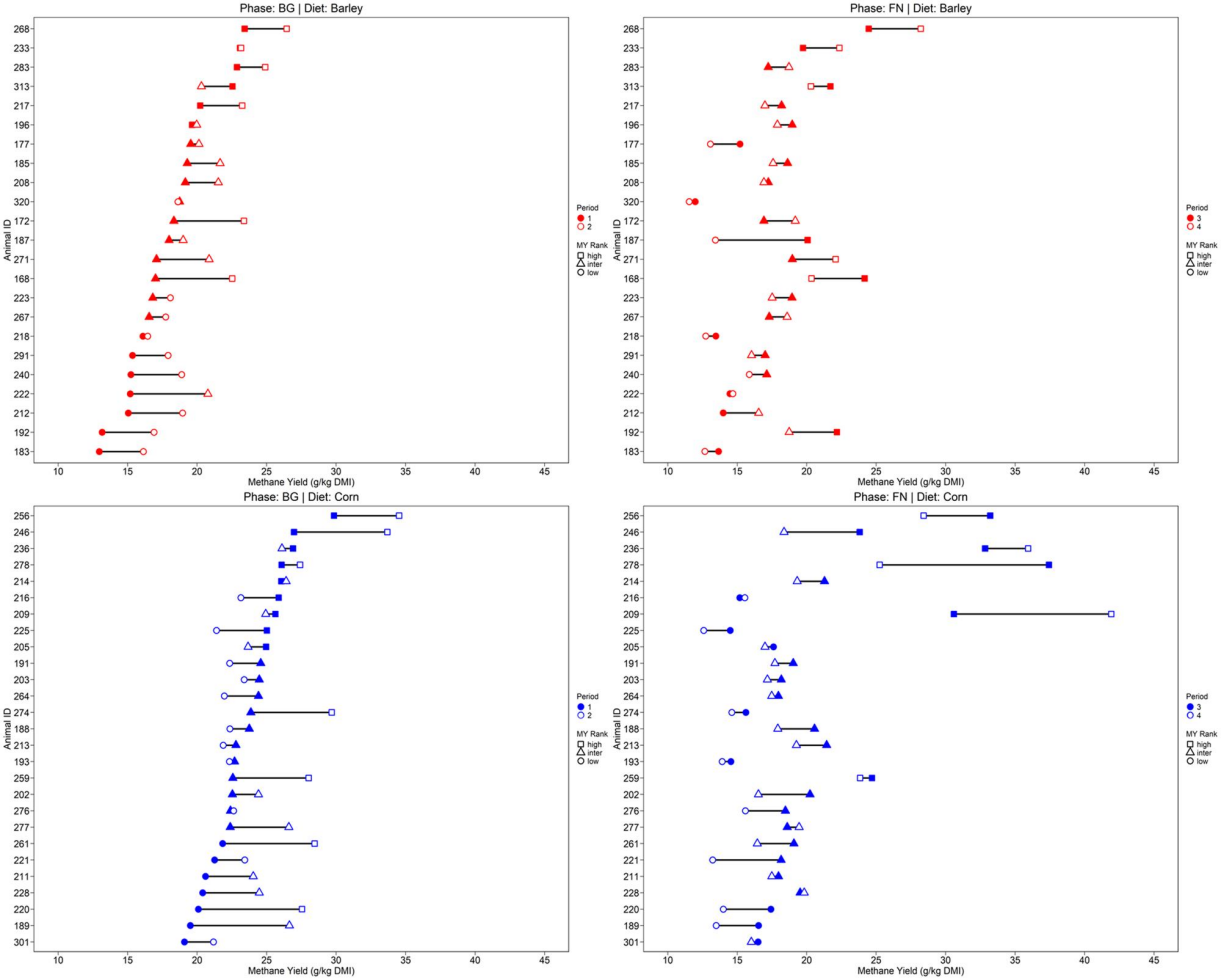
Methane yield (CH4Y; g/kg DMI) of steers in two periods of backgrounding (BG) and finishing (FN) phases fed barley or corn silage-based diets when ranked by methane yield rank (MYR) as Low, Intermediate, or High emitters using 0.5SD cut-offs on either side of the mean.

Steers were ranked by CH_4_Y and assigned a CH_4_Y grouping (**MYR**) for statistical analysis (Figure 1). This was achieved by determining the group mean for each silage source (BS or CS) and phase (BG or FN) and then calculating the cutoff as 0.5 standard deviations (**SD**) away from the mean in either direction. Steers were then categorized as low (< −0.5 SD from the mean), intermediate (within ± 0.5 SD from the mean), or high (> +0.5 SD from the mean) emitters for each period within the phase and overall for the phase. The changes in MYR for each steer from one period to the next (within BG and FN) and from one phase to the next (BG to FN) were examined. Rank changes were positive or negative depending on whether the animal went up or down a MYR, with a maximum of ±2 levels possible between the 3 MYR. Steers that did not change MYR from one measurement to the next received a 0 for rank change.

### Ruminal fermentation

During each phase, rumen digesta samples were collected via esophageal tube sampling, with sampling at the beginning of CH_4_ measurement for BG (d 1 of BG) and the middle of CH4 measurement for FN (d 21 of FN). The rumen digesta was strained through two layers of Pecap (mesh size 355 µm; Sefar Canada Inc., Ville St. Laurent, Canada), and 1 mL of filtrate was preserved with 0.2 mL of 5% (w/v) metaphosphoric acid for VFA analysis and 0.2 mL of 1% (v/v) H_2_SO_4_ for ammonia-nitrogen (**NH_3_-N**) analysis, respectively. Samples were stored at −20°C until analyzed. Rumen VFA were determined by gas chromatography (5890A Series Plus II, Hewlett-Packard Co., Palo Alto, CA, USA). The chromatograph featured a 30-m Zebron-free fatty acid phase fused silica capillary with an internal diameter of 0.32 mm and a film thickness of 1.0 μm (Phenomenex, Torrance, CA, USA). The concentration of NH_3_-N in rumen samples was analyzed using the phenol-hypochlorite method described by Broderick and Kang (1980).

### Calculations and statistical analysis

Data variance, distribution, normality, and homogeneity were examined using the UNIVARIATE procedure of SAS. The MYR data were analyzed using the PROC GLIMMIX function of SAS. The mixed model was used to analyze the fixed effects of MYR, silage source, and the interaction of MYR and silage source. The individual steer was the experimental unit and used as a random effect to account for individual variability. The covariance structure, compound symmetry, was used for repeated measures and chosen based on the lowest Akaike and Bayesian indicators. Means were estimated and separated using the LS Means function with a Tukey-Kramer adjustment to account for uneven group sizes. The fixed effects of period or phase were also included in the model for statistical analysis in which means were estimated for each period or phase by MYR × Silage source groupings for period 1 (**P1**), period 2 (**P2**), in BG and FN, as well as for BG overall (BG) and FN overall (FN) using pooled data for each phase.

The repeatability of the initial ranking of individual steers during each period (P1) was assessed by comparing P1 to their ranking in the subsequent period within the same phase, denoted as P2(P1). This comparison determined whether the ranking of individual steers was repeatable over time when fed the same diet. To assess changes in rankings over time, P2(P1) was also compared to the overall ranking of steers in P2. Similarly, the repeatability of rankings across phases was evaluated by comparing the initial rankings during the BG phase to the rankings of the same steers during the finishing phase, denoted as FN(BG). This comparison determined whether ranking consistency was maintained across production phases and dietary shifts. The FN(BG) rankings were also compared to the overall rankings in FN to assess whether the CH_4_ emission rankings of steers changed across phases. Significance was declared at *P *< 0.05.

Pearson correlations were conducted using the CORR procedure of SAS in which steers were grouped by silage source for each phase to determine the relationships between the CH_4_Y range (calculated as the absolute difference between the two-period CH_4_Y measurements of each phase) and other variables.

## Results

During the BG phase, there was no interaction between MYR and silage source for CH_4_Y, Ym, CH_4_, or DMI (*P *≥ 0.14; Table 2). Steers fed CS had greater CH_4_Y, Ym, and CH_4_, and lower DMI than those fed BS (*P *< 0.001). As expected, MYR significantly influenced CH_4_Y and Ym (*P *< 0.01), with differences observed among low, intermediate, and high steers in periods 1 (P1) and 2 (P2) across both diets. However, low and intermediate groups did not differ in P2(P1).

**Table 2:** The effects of methane yield ranking (MYR), silage source, and period on enteric methane (CH_4_) emissions and feed intake of steers ranked by methane yield (CH_4_Y) in period 1 (P1) and period 2 (P2) of backgrounding.

	Barley silage				Corn silage				*P* value		
Variable	Low	Intermediate	High	SEM	Low	Intermediate	High	SEM	MYR	Silage	MYR × Silage
**CH_4_Y, g/kg DMI**											
**P1**	14.7^C, y^	18.0^B, y^	22.0^A^	0.44	20.4^C, x^	23.3^B^	26.4^A^	0.41	<0.001	<0.001	0.36
**P2(P1)^1^**	18.0^B, z^	20.4^B, z^	23.0^A^	1.05	25.1^B, z^	24.1^B^	26.8^A^	0.97	<0.01	<0.001	0.20
**P2**	17.7^C, z^	20.5^B, z^	23.9^A^	0.54	22.4^C, y^	25.3^B^	29.9^A^	0.50	<0.001	<0.001	0.40
**Period *P* value**	<0.001	<0.01	0.24		<0.001	0.08	0.09				
**Ym (CH_4_, % GEI)**											
**P1**	4.41^C, y^	5.33^B, y^	6.52^A^	0.157	6.04^C, x^	7.05^B^	7.82^A^	0.145	<0.001	<0.001	0.28
**P2(P1)^1^**	5.36^B, z^	6.00^B, z^	6.76^A^	0.352	7.50^B, z^	7.21^B^	8.05^A^	0.356	<0.01	<0.001	0.27
**P2**	5.24^C, z^	6.10^B, z^	7.01^A^	0.187	6.72^C, y^	7.50^B^	8.98^A^	0.173	<0.001	<0.001	0.21
**Period *P* value**	<0.001	<0.001	0.29		<0.001	0.21	0.09				
**CH_4_, g/d**											
**P1**	138^C, y^	160^B, y^	200^A^	7.2	164^C, y^	190^B^	203^A, y^	6.6	<0.001	<0.01	0.14
**P2(P1)^1^**	166^z^	180^z^	202	9.2	203^z^	200	206^zy^	8.4	0.11	<0.01	0.25
**P2**	161^C, z^	179^B, zy^	216^A^	6.0	183^C, zy^	208^B^	228^A, z^	5.5	<0.001	<0.001	0.38
**Period *P* value**	<0.01	0.04	0.58		<0.01	0.17	0.05				
**DMI, kg/d^2^**											
**P1**	9.37	8.87	9.04	0.287	8.01	8.15	7.71	0.266	0.49	<0.001	0.31
**P2(P1)^1^**	9.22	8.85	8.74	0.266	8.05	8.32	7.75	0.246	0.21	<0.001	0.32
**P2**	9.10	8.69	9.03	0.268	8.18	8.22	7.68	0.248	0.45	<0.001	0.20
**Period *P* value**	0.62	0.81	0.75		0.85	0.86	0.97				

1 Period 2 means calculated for animals maintaining their period 1 MYR rankings.

2 DMI is the total intake of the total mixed ration and GreenFeed pellets.

*Note*: n = 50 where: barley silage n = 23 (Period 1 [P1] rankings; low n = 7, Intermediate n = 10, High n = 6; Period 2 [P2] rankings; Low n = 9, Intermediate n = 8, High n = 6), and barley silage n = 27 (P1 rankings; Low n = 7, Intermediate n = 11, High n = 9; P2 rankings; Low n = 11, Intermediate n = 9, High n = 7). Animals were sorted in ascending order based on methane yield, and assigned to ranks (MYR) as Low (less than -0.5SD from mean), Intermediate (within 0.5SD of mean), and High (more than +0.5SD from mean) emitters for each period.

A-C Means within a row with different letters differ (*P *< 0.05) for MYR main effect.

z-x Means within a column with different letters differ (*P *< 0.05) for Period effect.

Period effects were also evident during BG. For BS-fed low and intermediate steers, CH_4_Y and Ym were lower in P1 compared to P2 and P2(P1) (*P *< 0.001). For CS-fed low steers, CH_4_Y differed across all periods (*P *< 0.001), being lowest in P1 and highest in P2(P1). Across both diets, CH_4_ production differed by MYR in P1 and P2 (*P *< 0.001), but not in P2(P1) (*P *= 0.11). The BS-low steers had lower CH_4_ in P1 than in P2 and P2(P1) (*P *< 0.01). The CH_4_ was greater in P2(P1) compared to P1 for BS-intermediate and CS-low (*P *≤ 0.04), but not different from P2. The CS-high steers produced more CH_4_ in P2 compared to P1 (*P *= 0.05), with no difference between P2 and P2(P1). There were no MYR or period effects on DMI during BG (*P *≥ 0.21).

In the FN phase, a significant MYR × silage interaction affected CH_4_Y and Ym (*P *< 0.01; Table 3). The CS-High steers had the greatest emissions, while Low steers fed either silage had the lowest CH_4_Y and Ym across all periods. No interaction was observed for CH_4_ production or DMI in P2 (*P *≥ 0.22). However, an interaction was detected for DMI in P1 (*P *= 0.05), with BS-Low, BS-High, and CS-Low steers consuming more than CS-High but not differing from Intermediate steers. A similar pattern was observed for DMI in P2(P1) (*P *= 0.05), where both Low groups and CS-Intermediate steers had greater DMI than CS-High (*P *= 0.02), but did not differ from BS-Intermediate or BS-High.

**Table 3:** The effects of methane yield ranking (MYR), silage source, and period on enteric methane (CH_4_) emissions and feed intake of steers ranked by methane yield (CH_4_Y) in period 1 (P1) and period 2 (P2) of finishing.

	Barley silage				Corn silage				*P* value		
Variable	Low	Intermediate	High	SEM	Low	Intermediate	High	SEM	MYR	Silage	MYR × Silage
**CH_4_Y, g/kg DMI**											
**P1**	13.8^C, e^	17.9^B, cd^	22.1^A, b^	0.80	16.0^C, z, de^	19.3^B, z, bc^	30.4^A, a^	0.74	<0.001	<0.001	<0.001
**P2(P1)^1^**	13.5^C, c^	17.9^B, bc^	20.6^A, b^	1.36	14.6^C, zy, c^	17.5^B, y, bc^	29.0^A, a^	1.26	<0.001	<0.01	<0.01
**P2**	13.4^C, e^	17.7^B, cd^	22.7^A, b^	1.02	14.1^C, y, de^	17.9^B, y, c^	31.1^A, a^	0.97	<0.001	<0.001	<0.001
**Period *P* value**	0.90	0.90	0.61		0.02	<0.01	0.88				
**Ym (CH_4_, % GEI)**											
**P1**	3.99^C, e^	5.20^B, cd^	6.33^A, b^	0.249	4.69^C, z, de^	5.64^B, z, bc^	9.04^A, a^	0.232	<0.001	<0.001	<0.001
**P2(P1)^1^**	3.91^C, c^	5.18^B, bc^	5.87^A, b^	0.398	4.24^C, y, bc^	5.07^B, y, bc^	8.45^A, a^	0.497	<0.001	<0.01	<0.01
**P2**	3.88^C, e^	5.09^B, cd^	6.49^A, b^	0.291	4.10^C, y, de^	5.16^B, y, c^	9.09^A, b^	0.285	<0.001	<0.001	<0.001
**Period *P* value**	0.89	0.79	0.59		<0.01	<0.01	0.86				
**CH_4_, g/d**											
**P1**	157^C^	190^B^	247^A^	8.6	175^C^	200^B^	271^A^	8.0	<0.001	0.02	0.65
**P2(P1)^1^**	158^C^	190^B^	223^A^	10.9	172^C^	196^B^	251^A^	10.1	<0.001	0.07	0.58
**P2**	156^C^	195^B^	228^A^	8.9	160^C^	202^B^	264^A^	7.3	<0.001	0.03	0.22
**Period *P* value**	0.99	0.79	0.37		0.42	0.77	0.76				
**DMI, kg/d^2^**											
**P1**	11.39^a^	10.68^ab^	11.26^a^	0.407	10.93^a^	10.37^y, ab^	9.08^b^	0.379	0.07	<0.01	0.05
**P2(P1)^1^**	11.65^A, a^	10.66^AB, ab^	11.04^B, ab^	0.455	11.70^A, a^	11.18^AB, z, a^	9.10^B, b^	0.423	<0.01	0.22	0.02
**P2**	11.65^A^	11.02^A^	10.14^B^	0.452	11.36^A^	11.31^A, z^	8.88^B^	0.428	<0.001	0.26	0.24
**Period *P* value**	0.83	0.72	0.30		0.39	<0.01	0.98				

1 Period 2 means calculated for animals maintaining their period 1 MYR rankings.

2 DMI is the total intake of the total mixed ration and GreenFeed pellets.

*Note*: n = 50 where: barley silage n = 23 (P1 rankings; Low n = 6, Intermediate n = 11, High n = 6; P2 rankings; Low n = 7, Intermediate n = 11, High n = 5), and barley silage n = 27 (P1 rankings; Low n = 8, Intermediate n = 13, High n = 6; P2 rankings; Low n = 8, Intermediate n = 14, High n = 5). Animals were sorted in ascending order based on methane yield, and assigned to ranks (MYR) as Low (less than -0.5 SD from mean), Intermediate (within 0.5 SD of mean), and High (more than +0.5SD from mean) emitters for each period.

A-C Means within a row with different letters differ (*P *< 0.05) for MYR main effect.

a-c Means within a row with different letters differ (*P *< 0.05) for MYR × Silage interaction effect.

z-x Means within a column with different letters differ (*P *< 0.05) for Period effect.

Steers fed CS consistently produced more CH_4_Y, Ym, and CH_4_ than those fed BS (*P *≤ 0.02), except for CH_4_ in P2(P1) (*P *= 0.07). The DMI was greater for BS-fed steers than CS-fed steers in P1 (*P *< 0.01). For CH_4_ variables, all three MYR groups differed significantly from one another (*P *< 0.001). Intake was greater for low vs. high in P2 and P2(P1) (*P *< 0.01). In P2, intermediate DMI was similar to low, and both were greater than high in P2(P1), but intermediate did not differ from either low or high.

Period effects were present for CS-fed steers. The CH_4_Y and Ym were lower in P2 and P2(P1) compared to P1 for CS-intermediate, and Ym was lower for CS-low steers (*P *≤ 0.01). The production of CH_4_ for CS-low was lowest numerically in P2 compared to P1, but not different (*P *= 0.42) from P2(P1). For CS-intermediate steers, DMI increased in P2 and P2(P1) compared to P1 (*P *< 0.01).

When pooling periods within each phase, a MYR × silage interaction was found in FN for CH_4_Y and Ym (*P *< 0.001; Table 4). The CS-high steers had the highest emissions, followed by BS-High, both differing from all other groupings. Low and intermediate groups did not differ from each other within silage types, while BS-intermediate and CS-low were also similar. Silage source influenced CH_4_Y, Ym, CH_4_, and DMI during both BG and FN, with CS-fed steers showing higher emissions and lower intake than BS-fed steers (*P *≤ 0.03). A similar pattern was seen for CH_4_Y and Ym across the combined FN(BG) phase.

**Table 4:** The effects of methane yield ranking (MYR), silage source, and phase on enteric methane (CH_4_) emissions and feed intake of steers ranked by methane yield (CH_4_Y) in backgrounding (BG) and finishing (FN) phases.

	Barley silage				Corn silage				*P* value		
Variable	Low	Intermediate	High	SEM	Low	Intermediate	High	SEM	MYR	Silage	MYR × Silage
**CH_4_Y, g/kg DMI**											
**BG**	16.4^C, z^	19.4^B, z^	22.7^A^	0.46	22.3^C, z^	24.2^B, z^	28.1^A^	0.44	<0.001	<0.001	0.41
**FN(BG)^1^**	16.4^B, z^	17.1^B, y^	20.3^A^	1.66	17.2^B, y^	19.3^B, y^	25.5^A^	1.56	<0.01	0.04	0.45
**FN**	13.7^C, y, e^	17.6^B, zy, cd^	21.9^A, b^	0.68	15.1^C, x, de^	18.7^B, y, c^	31.4^A, a^	0.65	<0.001	<0.001	<0.001
**Phase *P* value**	0.02	0.04	0.28		<0.001	<0.001	0.23				
**Ym (CH_4_, % GEI)**											
**BG**	4.86^C, z^	5.76^B, z^	6.64^A^	0.139	6.70^C, z^	7.20^B, z^	8.43^A^	0.131	<0.001	<0.001	0.22
**FN(BG)^1^**	4.71^B, z^	4.95^B, y^	5.85^A^	0.486	5.02^B, y^	5.6^B, y^	7.55^A^	0.457	<0.01	0.03	0.38
**FN**	3.95^C, y, e^	5.08^B, y, cd^	6.30^A, b^	0.203	4.42^C, x, de^	5.43^B, y, c^	9.27^A, a^	0.194	<0.001	<0.001	<0.001
**Phase *P* value**	<0.01	<0.01	0.19		<0.001	<0.001	0.25				
**CH_4_, g/d**											
**BG**	147^C, y^	173^B, y^	207^A^	5.6	180^C^	195^B^	219^A^	5.3	<0.001	<0.001	0.20
**FN(BG)^1^**	185^B, z^	183^B, zy^	220^A^	13.2	188^B^	202^B^	235^A^	12.4	0.01	0.24	0.78
**FN**	157^C, zy^	191^B, z^	233^A^	8.2	167^C^	203^B^	270^A^	7.8	<0.001	<0.01	0.24
**Phase *P* value**	0.02	0.05	0.37		0.14	0.64	0.18				
**DMI, kg/d^2^**											
**BG**	8.98^y^	8.95^y^	9.11^y^	0.228	8.06^y^	8.08^y^	7.83	0.215	0.98	<0.001	0.64
**FN(BG)^1^**	11.22^z^	10.94^z^	10.86^z^	0.450	10.93^z^	10.81^z^	9.52	0.423	0.16	0.11	–
**FN**	11.52^A, z^	10.93^A, z^	10.67^B, zy^	0.416	11.07^A, z^	10.89^A, z^	8.83^B^	0.397	<0.01	0.03	0.10
**Phase *P* value**	<0.001	<0.001	0.04		<0.001	<0.001	0.22				

1 Means calculated for finishing by maintaining backgrounding MYR rankings.

2 DMI is the total intake of the total mixed ration and GreenFeed pellets.

*Note*: n = 50 where: barley silage n = 23 (BG rankings; Low n = 8, Intermediate n = 9, High n = 6, and FN rankings; Low n = 6, Intermediate n = 11, and High n = 6), and barley silage n = 27 (BG rankings; Low n = 9, Intermediate n = 12, High n = 6, and FN rankings; Low n = 8, Intermediate n = 14, and High n = 5 in FN). Animals were sorted in ascending order based on methane yield that was averaged across periods, and assigned to ranks (MYR) as Low (less than -0.5SD from mean), Intermediate (within 0.5SD of mean), and High (more than +0.5SD from mean) emitters for each period.

A-C Means within a row with different letters differ (*P *< 0.05) for MYR main effect.

a-c Means within a row with different letters differ (*P *< 0.05) for MYR × Silage interaction effect.

z-x Means within a column with different letters differ (*P *< 0.05) for Phase effect.

The CH_4_Y, Ym, and CH_4_ were different among MYR groups in BG and FN phases (*P *< 0.001), with low consistently lower than intermediate and both lower than high. For the FN(BG) phase, low and intermediate were similar to one another and both lower than high (*P *≤ 0.01). During FN, DMI was greater for low and intermediate than high (*P *< 0.01), while DMI did not differ across MYR in BG or FN(BG) (*P *≥ 0.16).

Phase comparisons revealed that CH_4_Y of CS-intermediate and Ym of both silage intermediate groups were lower in FN and FN(BG) than in BG (*P *< 0.01). The CH_4_Y and Ym of BS-low were greater in BG and FN(BG) than in FN (*P *≤ 0.02). For CS-low steers, CH_4_Y and Ym differed across all phases (*P *< 0.001), with FN being the lowest. The production of CH_4_ in BS-low was lowest in BG (*P *= 0.02), with no difference between FN(BG) and FN. In contrast, BS-intermediate steers had the greatest CH_4_Y in BG (*P *= 0.04) and the greatest CH_4_ in FN (*P *= 0.05), with FN(BG) not differing from either. Phase effects were present for DMI, with both silage types showing the lowest intake during BG for low and intermediate steers (*P *< 0.001). The BS-high steers had lower DMI in BG compared to FN(BG) (*P *= 0.04), with FN not different from either.

There were no interactions between MYR and silage on total or individual VFA, NH_3_-N, or feeding behavior during BG (*P *≥ 0.24; Table 5). Compared with BS-fed steers, those fed CS had a greater proportion of acetate, acetate:propionate ratio, and NH_3_-N concentration (*P *≤ 0.02) and lower proportions of propionate and valerate (*P *≤ 0.01). Low and intermediate steers had lower caproate levels than high steers (*P *< 0.01). No MYR or silage effects were observed on feeding behavior during BG (*P *≥ 0.11).

**Table 5:** The effects of methane yield ranking (MYR) and silage source on ruminal volatile fatty acid (VFA) proportions, ruminal ammonia (NH_3_-N) concentration, and feeding behaviour of steers ranked by methane yield (CH_4_Y) in backgrounding and finishing phases.

	Barley silage				Corn silage				*P* value		
Variable	Low	Intermediate	High	SEM	Low	Intermediate	High	SEM	MYR	Silage	MYR × Silage
**BACKGROUNDING**											
**Ruminal environment**											
**Total VFA, mM**	62.3	58.3	77.8	8.69	47.7	63.4	63.6	8.27	0.22	0.26	–
**Acetate, %**	68.4	70.1	68.1	1.67	73.0	72.7	71.7	1.59	0.66	0.01	0.81
**Propionate, %**	20.6	17.5	18.0	1.28	15.4	16.0	16.1	1.22	0.54	<0.01	0.24
**Butyrate, %**	7.93	9.24	10.31	9.168	8.55	8.1	9.6	8.741	0.25	0.60	–
**Isobutyrate, %**	0.78	0.88	1.07	0.096	0.91	0.97	0.93	0.092	0.59	0.33	–
**Valerate, %**	0.82	0.70	0.84	0.103	0.52	0.57	0.64	0.098	0.65	0.01	0.58
**Isovalerate, %**	1.37	1.50	1.49	0.134	1.57	1.58	1.11	0.128	0.22	0.76	–
**Caproate, %**	0.03^B^	0.10^B^	0.25^A^	0.048	0.06^B^	0.08^B^	0.21^A^	0.046	<0.01	0.85	0.79
**A: P^1^**	3.47	4.18	4.09	0.348	4.88	4.66	4.58	0.337	0.74	<0.01	0.30
**NH_3_-N, mM**	2.79	2.71	3.66	0.414	3.56	4.10	3.94	0.395	0.35	0.02	0.40
**Feeding Behavior**											
**Meal size, kg DM/meal**	0.97	0.99	0.98	0.060	0.85	0.91	0.94	0.057	0.65	0.11	–
**Meal frequency, per day**	8.72	8.75	9.19	0.475	8.92	8.27	7.94	0.447	0.76	0.18	–
**Meal duration, min**	17.8	17.0	16.3	1.55	18.5	19.2	19.5	1.45	0.98	0.11	–
**FINISHING**											
**Finishing**											
**Total VFA, mM**	92.6	88.2	86.3	9.41	61.0	80.2	81.9	8.99	0.66	0.06	–
**Acetate, %**	49.7^B^	53.0^AB^	56.6^A^	1.66	54.2^B^	53.5^AB^	57.0^A^	1.58	0.03	0.19	0.36
**Propionate, %**	37.3^A^	33.3^AB^	27.3^B^	2.68	30.6^A^	32.2^AB^	26.3^B^	2.56	0.04	0.19	0.48
**Butyrate, %**	7.26^B^	8.12^B^	11.39^A^	1.205	8.42^B^	8.89^B^	12.18^A^	1.184	<0.01	0.36	0.98
**Isobutyrate, %**	1.02^A, b^	1.18^AB, ab^	0.92^B, b^	0.107	1.55^A, a^	1.12^AB, b^	0.94^B, b^	0.103	0.01	0.07	0.01
**Valerate, %**	2.88^A^	2.04^B^	1.73^B^	0.214	2.60^A^	2.10^B^	1.61^B^	0.204	<0.001	0.52	0.69
**Isovalerate, %**	1.62	2.11	2.02	0.174	2.54	2.11	1.85	0.166	0.60	0.08	–
**Caproate, %**	0.26	0.21	0.05	0.046	0.08	0.09	0.14	0.044	0.31	0.09	–
**A:P^1^**	1.35^AB^	1.67^B^	2.45^A^	0.314	2.09^AB^	1.74^B^	2.45^A^	0.300	0.05	0.29	–
**NH_3_-N, mM**	10.13	10.51	8.48	1.281	7.72	10.30	9.10	1.224	0.30	0.52	–
**Feeding behavior**											
**Meal size, kg DM/meal**	1.85	1.71	1.97	0.116	2.03	1.83	1.59	0.111	0.25	0.77	–
**Meal frequency, per day**	6.24	6.15	5.52	0.385	5.21	5.78	5.24	0.368	0.31	0.08	–
**Meal duration, min**	11.1	10.2	10.5	0.81	12.2	11.3	10.3	0.78	0.34	0.31	–

1 Acetate to propionate ratio.

*Note*: n = 50 where: barley silage n = 23 (Backgrounding [BG] rankings; Low n = 8, Intermediate n = 9, High n = 6, and finishing [FN] rankings; Low n = 6, Intermediate n = 11, and High n = 6), and barley silage n = 27 (BG rankings; Low n = 9, Intermediate n = 12, High n = 6, and FN rankings; Low n = 8, Intermediate n = 14, and High n = 5 in FN). Animals were sorted in ascending order based on methane yield that was averaged across periods and assigned to ranks (MYR) as Low (less than -0.5 SD from mean), Intermediate (within 0.5 SD of mean), and High (more than +0.5SD from mean) emitters for each period.

A-B Means within a row with different letters differ (*P *< 0.05) for MYR main effect.

a-b Means within a row with different letters differ (*P *< 0.05) for MYR × Silage interaction effect.

In FN, there was an interaction between MYR and silage for isobutyrate proportion (*P *= 0.01), where CS-low steers had greater levels than BS-low, BS-high, CS-intermediate, and CS-high. No silage effects were observed for other VFA, NH_3_-N, or feeding behavior in FN (*P *≥ 0.06). High steers had greater acetate and acetate:propionate ratios (*P *≤ 0.05), and lower proportions of propionate and isobutyrate (*P *= 0.04) than low steers. Low and intermediate steers had lower butyrate than high steers (*P *< 0.01), and valerate was greater in intermediate and high than low (*P *< 0.001).

Across periods in BG, 44% of steers remained in the same MYR from P1 to P2, while 15% of low-ranked and 13% of high-ranked steers shifted to the opposite ranking (Table 6). In FN, no low-ranked steers were reranked as high, though 8% of high steers were reranked as low. Rankings were more stable during FN than BG, with 80% of steers maintaining the same rank from P1 to P2. Across phases, 58% of steers retained their MYR, 7% of low steers were reranked as high, and 20% of high steers were reranked as low. Among BS-fed steers, 65%, 78%, and 57% remained in the same rank during BG, FN, and across phases, respectively. For CS-fed steers, the corresponding values were 26%, 82%, and 59%.

**Table 6:** Methane yield group (MYG) rank changes between periods and phases overall and based on diet.

	All diets				Barley				Corn			
Starting rank	L	I	H	SUM	L	I	H	SUM	L	I	H	SUM
**Percent of animals—rank change from backgrounding period 1 to period 2**												
**N**	14	21	15	50	7	10	6	23	7	11	9	27
**+2**	15	.	.	4	0	.	.	0	29	.	.	7
**+1**	28	19	.	16	14	20	.	13	42	18	.	19
**0**	57	33	47	44	86	50	67	65	29	18	33	26
**−1**	.	48	40	32	.	30	33	22	.	64	45	41
**−2**	.	.	13	4	.	.	0	0	.	.	22	7
**Percent of animals—rank change from finishing period 1 to period 2**												
**N**	14	24	12	50	6	11	6	23	8	13	6	27
**+2**	0	.	.	0	0	.	.	0	0	.	.	0
**+1**	21	4	.	8	17	9	.	9	25	0	.	7
**0**	79	83	75	80	83	82	67	78	75	85	83	82
**−1**	.	13	17	10	.	9	17	9	.	15	17	11
**−2**	.	.	8	2	.	.	17	4	.	.	0	0
**Percent of animals—rank change from backgrounding (period 1) to finishing (period 1)**												
**N**	14	21	15	50	7	10	6	23	7	11	9	27
**+2**	7	.	.	2	14	.	.	4	0	.	.	0
**+1**	43	14	.	18	29	20	.	17	57	9	.	19
**0**	50	67	53	58	57	60	50	57	43	73	56	59
**−1**	.	19	27	16	.	20	50	22	.	18	11	11
**−2**	.	.	20	6	.	.	0	0	.	.	33	11

Notes: Animals were sorted in ascending order based on methane yield, and assigned to groups (MYG) as Low (less than -0.5 SD from mean), Intermediate (within 0.5 SD of mean), and High (more than +0.5 SD from mean) emitters for each period. Period rankings n = 50 were: barley is Low n = 7, Intermediate n = 10, and High n = 6 in Period 1 of BG and Low n = 6, Intermediate n = 11, and High n = 11 for Period 1 of FN; corn is Low n = 7, Intermediate n = 11, and High n = 9 for Period 1 of BG, and Low n = 8, Intermediate n = 13, and High n = 6 for Period 1 of FN.

z Rank change is the number of MYGroups the animal changes from one period or phase to the next.

No strong correlations were found between CH_4_Y and feeding behavior during BG (*r *≤ 0.31; *P *≥ 0.13; Table 7). In FN, CH_4_Y variability was moderately to strongly correlated with CH_4_Y and Ym in both silage groups (*r *≥ 0.47; *P *≤ 0.02). For CS-fed steers, CH_4_Y range was positively correlated with CH_4_ (*r *= 0.44; *P *= 0.02) and negatively correlated with DMI (*r* = –0.61; *P *< 0.001).

**Table 7:** Pearson correlation coefficients (r) of the relationship between the range of methane yield (CH_4_, g/kg DMI) during backgrounding and finishing phases with methane (CH_4_) measurements and feeding behavior by phase of steers fed barley silage or corn silage diets.

Variable^z^	Barley silage	Corn silage
**BACKGROUNDING**		
**Methane measurements**		
**CH_4_Y, g/kg DMI**	**−**0.09	0.30
**CH_4_, g/d**	0.01	0.18
**Ym (CH_4_, %GEI)**	**−**0.11	0.24
**Feeding behavior**		
**DMI, kg/d**	0.26	**−**0.12
**Meal size, kg DM/meal**	0.31	0.06
**Meal frequency per day**	**−**0.27	**−**0.03
**Meal duration, min**	0.12	0.11
**FINISHING**		
**Methane measurements**		
**CH_4_Y, g/kg DMI**	0.47^†^	0.67*
**CH_4_, g/d**	0.36	0.44^†^
**Ym (CH_4_, %GEI)**	0.48^†^	0.69*
**Feeding behavior**		
**DMI, kg/d**	**−**0.23	**−**0.61*
**Meal size, kg DM/meal**	**−**0.01	**−**0.32
**Meal frequency per day**	**−**0.07	**−**0.22
**Meal duration, min**	0.14	0.05

z Variables were mean values by phase (backgrounding or finishing).

† *P *< 0.05;

* *P *< 0.001. Correlation coefficient is equal to √R^2^.

*Note*: Range of methane yield was calculated as the absolute value difference in CH_4_Y between period 1 and period 2 measurement where barley n = 23, and corn n = 27.

## Discussion

Efforts to reduce enteric CH_4_ emissions from cattle are increasingly targeting the selection of animals with inherently low CH_4_ production, as this trait offers a heritable and potentially permanent path toward lowering emissions. Moderate heritability estimates (h^2^ = 0.25–0.42) support the feasibility of genetic selection (Hayes et al. 2016; Manzanilla-Pech et al. 2016; Brito et al. 2018; Souza et al. 2024); however, identifying reliable low emitters requires confidence that CH_4_Y measurements are consistent across time and diets. This study builds on previous work (Beauchemin et al. 2022; Vargas et al. 2025) by evaluating the variability and repeatability of CH_4_Y in feedlot steers across two production phases, BG and FN, and two diets differing in silage source (barley vs. corn).

### CH_4_Y variability and repeatability

The consistency of CH_4_ emissions in cattle fed high-grain diets has been previously examined (Beauchemin et al. 2022), with a between-animal coefficient of variation of 17.3% for cattle from a single source and fed a common diet. Beauchemin et al. (2022) measured CH_4_ production in cattle fed a BG diet over a 6-wk period and found that the ranking of some animals changed over time; however, there was moderate repeatability (0.56–0.57) between measurement periods. Recently, Vargas et al. (2025) investigated whether growth-promoting implants or a diet change between BG and FN influenced CH_4_ ranking of the animals. Those authors reported that the ranking of animals for CH_4_ changed between phases, implying that phenotyping animals for CH_4_ production needs to be performed independently during each production phase (Vargas et al. 2025). The present study builds on those previous studies by examining the CH_4_Y of steers within and across high forage and high grain diets.

As intended, the ranking of steers by CH_4_Y resulted in low steers having lower CH_4_, CH_4_Y, and Ym compared to high steers during each period and phase. However, the difference in CH_4_Y between low and high steers in BG based on P1 ranking decreased during later periods (ie, P2(P1)) and across phases (ie, P2(P1) or FN(BG)). For example, in P2 of BG, the difference in CH_4_Y between steers identified as low and high for P1 was 4.2% and 18.8% less for BS and CS, respectively, than that of steers ranked as low and high in P2 with no regard for their ranking in P1. Similarly, for FN, the difference in CH_4_Y between low and high steers was 6.6% and 5.1% less for BS and CS, respectively, when comparing P2(P1) with P2 rankings. The same trend, however, to a greater extent, was observed when examining CH_4_Y in FN using FN(BG) rather than FN ranking, where the difference between high and low was reduced by 18.2% for BS and 19.4% for CS. These results suggest that the difference between low and high-ranked steers is directly influenced by the period during which they are measured for emissions, indicating the need for repeated measurements over time, even when the diet remains unchanged. Furthermore, the variability between high- and low-ranked emitters appear to differ according to diet, as the variability was greater for animals fed CS compared with BS.

Differences in DMI did not account for differences in MYR among steers during BG, suggesting a possible role of other mechanisms such as genetics (Ryan et al. 2025), digestion efficiency (Duthie et al. 2017), microbial communities (Smith et al. 2022), feeding behavior, and physiology. In contrast, during FN, steers with higher CH_4_Y had lower DMI, which agrees with previous studies utilizing high-concentrate diets (Ryan et al. 2022), but not others (Beauchemin et al. 2022). The differing influence of DMI on CH_4_Y for BG and FN may reflect the greater variation in DMI during FN than during BG.

During BG, the differences in MYR were not associated with differences in ruminal VFA proportions. The one exception was an increase in caproate for high compared to intermediate and low emitters. Caproate is a hydrogen sink and has been shown to increase in the CH_4_-inhibited rumen when using inhibitors (ie, 3-nitrooxyproanol; Guyader et al. 2017). The greater caproate proportions in high emitters is consistent with excess metabolic hydrogen produced in the rumen and greater production of CH_4_. Although a significant difference was found for caproate, the minimal contribution of this VFA (less than 0.3% in this study) to total VFA concentration diminishes its role as a hydrogen sink. Beauchemin et al. (2022) found that total VFA concentration decreased with decreasing CH_4_Y, suggesting a reduction in OM fermentation in the rumen of low emitters. The reduction in OM fermentation could be due to an increased rumen passage rate, which is known to decrease CH_4_ production (Pinares-Patino et al. 2003). The lack of differences in most VFA proportions between MYR groups in the present study may indicate that the collection method (esophageal sampling), frequency (once in a single day), and timing of rumen fluid sampling were insufficient when making these comparisons. The potential variability in the location of rumen fluid collection when using esophageal sampling could increase sample variability, reducing the ability to detect treatment differences. This is an area of research that requires further examination, with more frequent ruminal fluid sampling to determine possible diurnal differences in VFA proportions across different MYR. Alternatively, lower CH_4_ production, with no change in VFA proportions, could imply that other factors not measured in this study, including the rumen microbiome, VFA absorption efficiency, and host physiology between animals of different MYR may influence CH_4_Y.

During FN, steers categorized as low emitters had a greater proportion of propionate and a lower proportion of acetate compared with high emitters. This shift in VFA proportions is consistent with reduced CH_4_ production, as propionate acts as an alternative hydrogen sink to CH_4_ within the rumen (Kobayashi 2010; Pereira et al. 2022). In contrast to the present study, Beauchemin et al. (2022) observed no difference in VFA proportions between beef heifers differing in MYR when fed a high grain FN diet. The authors suggested that the lack of differences in VFA among rankings may have been due to the high-concentrate diet fed, where propionate proportions were high for all animals (≥ 37.4 mol/100 mol). In our study, the propionate proportions for all rankings were lower than those reported by Beauchemin et al. (2022), which may explain the contrasting results in the two studies.

### Consistency of MYR across time and diets

Diet is known to affect DMI, VFA proportions, and CH_4_ emissions; thus, identifying low-emitting cattle may be challenging across dietary changes. Furthermore, no previous studies have examined the impact of silage sources on the consistency of ranking of cattle for CH_4_Y. In our study, CH_4_ emissions of cattle during each period (P1, P2) and phase (BG, FN) were compared to assess the change in rank from one period or phase to the next, as affected by silage source.

Within BG, the CS-low steers in P1 exhibited a CH_4_Y that was 12.1% higher in P2 than the animals ranked low in P2, indicating that an animal’s ranking depended on the measurement period. The CS-fed steers had the lowest percentage of steers (26%) that did not change ranking during BG, compared to BS-fed steers (65%). Additionally, CS-low steers had a greater likelihood of extreme rank change (±2 levels) than CS-high steers, at 71% and 67%, respectively. Regardless of silage source, the likelihood of rank change during BG was 8%, 48%, and 44% for changes of 2, 1, and 0 ranks, respectively. Therefore, ranking animals over 2 wks in the BG phase identified high emitters more accurately than low or intermediate emitters. These results indicate that a 2-wk CH_4_ measurement period using the GEMS may not reflect emissions over time in steers fed a high-forage diet. The results underscore the need to account for changes in average daily CH_4_ production over multiple weeks.

During FN, the reranking of cattle by CH_4_Y in P2 did not alter mean CH_4_Y from the initial rankings in P1, suggesting that when fed a high-concentrate diet, the need for repeat measurements for ranking cattle may be diminished. This is evidenced by the relatively small percentage of animals during FN that changed rank (20%), regardless of silage source. The only source of extreme rank change for this phase was observed in BS-high steers, with 17% of all BS-fed steers changing rankings. Beauchemin et al. (2022) found that cattle fed a high-grain diet (silage at 8% of DM), ranked as low and very low (bottom 25%), were misidentified 15%–23% of the time using the GEMS system from one period to the next. In that study, cattle were ranked into five groups (very low; 0%–10%, low; 10%–25%, intermediate; 26%–74%, high; 75%–89%, and very high; 90%–100%) instead of the three groups used in the present study, which may have contributed to the differences in rank changes observed. In the current study, the differentiation between MYR was maintained for P1, P2(P1), and P2 in FN, demonstrating the ability to accurately identify Low emitters during a single 2-wk period when animals were fed a high-grain diet.

The most significant changes in mean MYR were when the ranking from BG was compared to that in FN. The BS-low and CS-low were 19.7% and 13.9% greater for FN(BG) compared to FN, indicating that some low emitters during BG had comparatively greater CH_4_ emissions in FN. Regardless of silage source, the likelihood of changing rank from BG to FN was 8%, 34%, and 58% for a change of 2, 1, and 0 rankings, respectively. A recent study by Vargas et al. (2025) examined MYR when steers were transitioned from a corn-based BG diet containing 43% forage DM to an FN diet containing 20% forage DM and reported that 50%–65% of steers changed MYR. This trend also aligns with Goopy and Hegarty (2004), who observed a lack of consistent ranking of cattle CH_4_ yield when diet types were alternated between high forage and high grain. Beauchemin and McGinn (2005) found that CH_4_Y was reduced from BG to FN by 38% and 64% for cattle fed high-grain diets that included BS and CS, respectively. That study reported a more dramatic reduction in CH_4_ during FN compared to the present study (4%–20% reduction for BS and 11%–48% for CS), which could be linked to differences in silage inclusion between their study (9% of DM) and the present study (15% of DM).

When assessing CH_4_Y during FN between initial ranking (FN(BG)) and ranking during FN (FN), the low and intermediate animals were similar and less than the high animals, indicating that a single measurement period is more likely to accurately identify high emitters than low emitters. Additionally, these results suggest that a change in diet does not result in similar variations between high, intermediate, and low emitters. The ability to accurately select low emitters relies on measurements conducted over multiple periods when fed high forage diets and should be repeated when the diet changes.

### Factors contributing to CH_4_Y variability

The relationship between variability in CH_4_Y and other production traits varied across diets and production stages, indicating differences in the stability of these traits throughout feedlot production. During BG, the weak correlations between CH_4_Y range (ie, variability across periods) and CH_4_ measurements or feeding behavior imply that early-stage CH_4_Y variability is not strongly linked to the traits measured in this study. This instability could stem from environmental, physiological, and microbial factors during early growth, when cattle primarily consume high-forage diets. These observations align with those of Beauchemin et al. (2022), who found that differences in feeding behavior were not related to CH_4_ variation between low and high emitters during BG. Consequently, MYR rankings in the BG phase may be somewhat temporary, especially for low emitters whose emissions tend to fluctuate more on forage-rich diets.

In contrast, during FN, variability in CH_4_Y was more strongly positively associated with CH_4_ and Ym, especially under CS diets. This suggests that steers with higher emissions or less efficient energy use (higher Ym) also showed greater fluctuations in CH_4_Y over time. Conversely, low emitters during FN tended to have a narrower range of CH_4_Y, indicating they were more consistent during this phase. These findings align with those of Ryan et al. (2022), who observed greater repeatability of CH_4_ traits during FN, and support the findings of Beauchemin et al. (2022), who demonstrated that low-emitting animals consistently maintained low emissions despite similar intake and performance to high-emitting animals. This greater consistency makes low emitters easier to identify during FN, especially on high-energy diets that lessen diet-related variation.

Previous studies (Nkrumah et al. 2006) found moderate correlations between feeding behavior and CH_4_ production. However, in the present study, these traits did not explain the variation in CH_4_Y. Overall, the findings suggest that variability in CH_4_Y is more closely related to phase and dietary effects than to feeding behavior patterns. Additionally, measurements taken during late stages under FN diet conditions may be the most reliable for identifying animals with consistently low CH_4_ emissions.

### Silage source

Methane production, yield, and Ym were greater for CS compared to BS during each period and phase of this experiment, which aligns with the BG phase of Beauchemin and McGinn (2005); however, during the FN phase, those authors observed the opposite response. As previously stated, this inconsistency could be due to the differences in the inclusion rate of silage during FN in their study (15% of DM) compared to the present study (9% of DM). The interaction of MYR and silage source was absent during BG, indicating that the relative differences between the ranking groups were not affected by silage type. However, during FN, MYR × silage source interactions were found for CH_4_ and Ym, where BS-high cattle had 27.3% lower emissions than CS-high cattle, suggesting that silage source had a greater impact on high rather than low- or intermediate-ranked steers. The results suggest that feeding CS results in greater CH_4_ emissions, compared to BS in high and low forage diets. The highest emitters fed CS during BG had significantly greater emissions than the highest emitters fed BS. This information suggests that selecting high emitters requires the animals to be fed a consistent diet with minimal changes in ingredient composition. In our study, even though animals were fed a high-concentrate FN diet, a change in the silage source (included at 15% of the diet DM) affected CH_4_ production.

Steers fed BS had greater DMI and lower CH_4_ than CS-fed steers throughout BG and FN, suggesting that differences in CH_4_ production were not a result of differences in DMI. In contrast, Beauchemin and McGinn (2005) found that DMI was less for BS than CS diets during BG when the silage source constituted 70% of the diet on a DM basis. This difference between studies may have occurred because barley grain was used for all diets in the present study, whereas Beauchemin and McGinn (2005) matched the grain source to the silage source for each diet. In the present study, differences in the NDF (39.7%, CS; 38.6%, BS) and starch concentration (40.6%, CS; 42.9%, BS) of the diets were small; although DM digestibility corn silage was expected to be greater than that of compared to barley silage (Johnson et al. 2020), which may have contributed to the observed differences in feed intake (Allen 2014; Riaz et al. 2014). Greater DMI of BS-fed steers during BG without a difference in total VFA concentration in the rumen suggests that the fermentability and digestion of CS were greater, allowing for improved nutrient supply and reducing the need for feed to meet requirements.

During BG, BS resulted in lower acetate and greater propionate and a lower A:P ratio compared with CS, possibly a result of differing starch concentration or processing index in the silage source, which favors propionate production (Bannink et al. 2008; Beauchemin et al. 2009). Alternatively, the BS diet was slightly higher in barley grain proportion than was the CS diet (26.48 vs. 22.07%). In agreement with the present study, similar shifts in VFA concentrations were reported when feeding BS or CS in high-forage diets (Beauchemin and McGinn 2005; Sutherland et al. 2021). Although some research has observed similar shifts in VFA with increasing CS inclusion at the expense of BS (Benchaar et al. 2014), where the difference in starch content from one treatment to another was up to 14%, compared to 2% in the present study. These small differences in nutrient composition of the BS and CS diets in the present study likely reduced the potential for large differences in rumen fermentation. Additionally, it has been shown that increased propionate within the rumen is associated with lower CH_4_ production, as seen in the present study for BS-fed steers throughout BG. Silage source influenced DMI and ruminal fluid VFA concentrations during BG to a greater extent than in FN, likely due to the greater inclusion of silage (70%) in the BG diet compared to the FN (15%). In the present study, when feeding BS, there was a shift in VFA proportions toward alternative hydrogen sinks and less methanogenesis, which was less prominent when feeding CS. This shift was more readily observed when feeding a high forage compared to a high concentrate diet.

## Conclusion

This study examined the consistency of MYR in beef steers across different diet types and silage sources, both within and between production phases for feedlot cattle. We observed that 66%, 20%, and 42% of steers changed MYR during BG, FN, and from BG to FN, respectively. Notably, Low emitters were more likely to change rank if fed CS than if fed BS, indicating greater variability of CH_4_Y during BG and the influence of silage source. Methane yield was consistently higher for CS than BS, regardless of diet type. This may have resulted from BS steers having a greater DMI and a rumen fermentation profile that favored lower CH_4_ production during BG. VFA profiles revealed limited differences across emitter rankings, suggesting that the differences between low and high emitters may not be linked to differences in ruminal fermentation. The results of this study suggest that low emitters are less consistent in their CH_4_ ranking when fed high-forage diets.

Overall, the inconsistency in MYR indicates that identifying low-emitting steers requires repeated measurements over time and across different diets. Further understanding of the factors that cause variability in CH_4_ emissions data, including GEMS usage by individual animals and diet composition, is warranted to provide the clarity needed to identify CH_4_ mitigation solutions that reduce greenhouse gas emissions in ruminant livestock industries.

## Data Availability

The raw data supporting the conclusions of this article will be made available by the corresponding author upon reasonable request.

## Abbreviations

ADG: average daily gain
BG: background
BS: barley silage
BW: body weight
CH_4_: methane
CH_4_Y: methane yield
CP: crude protein
CS: corn silage
DM: dry matter
DMI: dry matter intake
DOF: days on feed
FN: finishing
FN(BG): finishing using backgrounding rankings
GEI: gross energy intake
GEMS: GreenFeed emissions monitoring system
High: high ranked methane yield group
Intermediate: intermediate ranked methane yield group
Low: low ranked methane yield group
MYR: methane yield rank
NDF: neutral detergent fiber
NH_3_-N: ammonia-nitrogen
OM: organic matter
P1: period 1
P2: period 2
P2(P1): period 2 using period 1 rankings
SD: standard deviation
TMR: total mixed ration
Ym: methane energy as a percentage of gross energy intake
